# Perceived costs and benefits of companion dog keeping based on a convenience sample of dog owners

**DOI:** 10.1038/s41598-025-85254-1

**Published:** 2025-01-21

**Authors:** Laura Gillet, Borbála Turcsán, Eniko Kubinyi

**Affiliations:** 1 Department of Ethology, ELTE Eötvös Loránd University, Budapest, Hungary; 2 MTA-ELTE Lendület “Momentum” Companion Animal Research Group, Budapest, Hungary; 3 ELTE NAP Canine Brain Research Group, Budapest, Hungary

**Keywords:** Dog ownership, Costs, Benefits, Well-being, Dog-owner relationship, Human behaviour, Animal behaviour

## Abstract

The increasing trend of dog ownership is often linked to its perceived benefits for human physical and mental well-being. However, the psychological and practical demands of caring for a dog can significantly impact the owner’s quality of life and the long-term success of the dog-owner relationship. This study aimed to provide a comprehensive overview of both the advantages and disadvantages of dog ownership, as perceived by a convenience sample of Hungarian dog owners (N = 246), who were assumed to be generally satisfied with their dogs. The study employed both quantitative (a Cost/Benefit scale consisting of 33 neutrally-phrased statements rated on a seven-point scale, from − 3 to + 3) and qualitative methods (two open-ended questions). Quantitative results showed that owners rated the short lifespan of dogs as the most negative aspect, while the belief that dogs brighten their lives was rated most positively. On average, positive statements were rated significantly higher (2.06) than negative ones (−0.66), supporting the prediction from social exchange theory that dog owners in a voluntary sample tend to perceive more advantages than disadvantages in dog ownership. Qualitative findings revealed six ‘benefit’ and three ‘cost’ themes. 61% of owners considered the meaningful relationship with their dog as the greatest benefit, frequently mentioning the dog’s constant presence, love, and support. Additionally, 15% highlighted the relationship with another species and the dog’s intrinsic qualities, indicating the biophilia effect of dog ownership. Regarding the costs, 95% of respondents identified financial, particularly health-related expenses, as the most significant drawback, and only 4–5% mentioned emotional or practical burdens. In the quantitative data, principal component analysis revealed three core components of the dog ownership experience: (1) emotional, physical, and social benefits, (2) negative emotions and practical challenges, and (3) time and emotional commitment. Overall, the results suggest clear costs and benefits, though some aspects, such as daily care, holiday arrangements, and training, were seen as both beneficial and disadvantageous, depending on the owner. Even within a convenience sample, the diversity in dog and owner characteristics was sufficient to explain why certain facets of dog ownership are experienced differently.

## Introduction

The pet dog population has been growing worldwide, especially during the COVID-19 pandemic^1^. In 2023–24, the number of households owning at least one dog is estimated to be around 65.1 million in the United States and around 104 million in Europe^2,3^. Most prospective owners expect positive effects from dog ownership, such as getting more physical exercise, making new friends and improving their mood^4,5^, while underestimating certain costs, like the financial costs or the amount of patience required to handle a dog^6^. Culture, as conveyed by mainstream media, can be argued to contribute to this biased perception of dog ownership, favouring its positive aspects for both people and dogs^7,8^.

Although dog ownership can have positive effects on human health, these are far from being universal. Research has revealed conflicting or equivocal results regarding a wide range of outcomes, including depressive symptoms, physical health, loneliness, blood pressure, physical activity and general well-being^9–14^. Overall, systematic reviews cannot conclude that pets, in general, have a positive influence on mental and physical well-being, mostly due to methodological limitations and the heterogeneity of both pets and the pet owner population^15–19^.

Why would some dogs worsen the health of their owners? Apart from accidents, bites and other health hazards, one hypothesis is that dog ownership comes with its emotional challenges, which may elicit negative emotions such as guilt, anger, sadness or worry. This might, in turn, increase the general levels of stress and depression of owners^9,20,21^. For instance, these challenges arise when the dog is sick or aging^22–24^, or when the owners feel like they are incapable of meeting their dog’s needs. Dealing with the passing of the dog can also be an emotional hardship^25^. Likewise, unwanted pet behaviours, such as barking, aggression, separation-related problems, and attention-seeking behaviours, may irritate or distract household members^26^. Besides these emotional challenges, other costs or disadvantages of dog ownership are just as important to consider. For instance, having a dog often means more practical burdens (e.g., pet supply shopping, holiday care, living arrangements, expenses)^26–29^. When these tangible responsibilities impact the daily life of the owner negatively (e.g., missed social opportunities because the dog had to be looked after, conflicts with the neighbourhood due to the dog’s behaviour), they might turn into a psychological burden^30^. Dogs also represent a health risk for the general population (e.g., zoonoses, biting, allergies) and have an ecological ‘paw print’ that should not be overlooked^31–34^.

If owning a dog can be so costly, the question arises of why more and more people decide to do it anyway. According to the Social Exchange Theory (SET)^35^, humans decide to develop and maintain relationships with each other by weighing the costs and benefits of these relationships. The perceived benefits of the relationship should equal or outweigh the costs for it to last. Following the hypothesis first formulated by Netting et al.^36^, several researchers have then suggested that the SET theory could be applied to human-pets relationships, too (e.g.,^9,37^). Therefore, in line with the SET, it is expected that owners keep their dogs because it provides them with perceived benefits that outweigh all costs. If the perceived costs are too high, people might instead choose not to acquire a dog, or to relinquish their current dog. Reasons given for not owning a dog can be both emotional and pragmatical, and often relate to a lack of time, housing limitations and anticipatory grief^27,38–40^. As for relinquishment, it affects thousands of dogs each year across the globe. This phenomenon, therefore, raises a serious animal welfare issue. Behavioural problems, mainly aggressivity and destructiveness, constitute the leading animal-related reasons for dog rehoming^41^. Human-related reasons are usually due to a change in the owner’s circumstances (e.g., moving, family issues, illness)^41^, but gaps between expectations and reality can also lead to higher costs than anticipated^42^. Prospective owners with more knowledge about dog care have more realistic expectations and are less likely to relinquish the dog after adoption^43,44^.

Yet, some owners are willing to pay a high price to maintain their relationship with their companion, sometimes putting their own health or professional career at risk to prioritise the dog’s needs^45,46^. According to Sutcliffe et al.^47^, people make different cost–benefit trade-offs depending on the quality of the relationship. Relationships with closer, more intimate social partners require more investment to be maintained, but they provide greater emotional support than more distant partners. In the case of dog owners, different characteristics of the dog-owner relationship, such as attachment or perceived emotional closeness, have indeed been found to influence the effects of dog ownership on the psycho-social well-being of both adults and children (e.g., depression, anxiety, self-esteem, social development)^20,48^. Thus, one hypothesis is that owners who invest more time and money in their dog counterbalance these costs with the perceived benefits they gain from the dog’s presence. Of course, as motives to acquire a dog are diverse^4^, the perception of what counts as a cost and what counts as a benefit in the dog–human relationship may vary from owner to owner (see examples in^49^).

While investigating the potential benefits of dog ownership is of great relevance in a social context in which an increasing number of people suffer from mental health issues, such as depression and anxiety^50^, its costs should not be overlooked either. However, comprehensive approaches to this topic are rare. How do dog owners assess the different facets of dog ownership (i.e. all the potential experiences, responsibilities, and consequences that come with having a dog) in the light of their own experience? Which facets do they consider the most costly, and which the most beneficial?

Previous studies addressing such research questions have had several limitations. Firstly, they have mostly focused on specific positive outcomes, such as health-related outcomes, well-being, and dog-owner relationship satisfaction, while rarely exploring other potential benefits of dog ownership (e.g., contribution to self-growth, improving family cohesion, contribution to animal welfare through the adoption of rescued dogs) or addressing its negative outcomes^51–54^. Secondly, the scales used to assess the costs and benefits of dog ownership usually reflect the researchers’ a priori categorization of certain aspects of having a dog as (dis)advantages (e.g.,^55^). For example, the Monash Dog Owner Relationship Scale (MDORS)^56^, which includes nine items relative to a factor labelled as the *Perceived Costs* of dog ownership, has been widely used in human–dog relationship research (e.g., in^57,58^). However, the phrasing of these nine items, using words such as ‘annoying’, ‘too much’ or ‘bothers’, likely encourages respondents to perceive them as costs, introducing a potential framing effect^59^. A last limitation of previous findings is that they often apply to specific subgroups of dogs and/or owners, such as families with children and young mothers^28,60,61^, elderly owners^27,62,63^, owners with diagnoses of mental health issues^64^, and owners of specific dog breeds (e.g., Samoyed, brachycephalic dogs)^29,65^ or of dogs with behavioural problems^66,67^. Some studies have also focused on the difficulties of having a dog in times of crisis, for instance, during the COVID-19 pandemic^10,26,68^ or in case of financial hardship^42^.

### The present study

The main aim of the present study is to explore how the different facets of dog ownership are assessed by a convenience sample of dog owners, who are assumed to be overall satisfied with their dogs. We decided to use a mixed-method approach, combining quantitative and qualitative data.

First, we developed a new scale to quantify the perceived valence of different facets of dog ownership. This quantitative tool was specifically designed to avoid a pre-categorisation of the scale items into costs on one hand, and benefits on the other hand. In particular, participants were asked to rate various neutrally-phrased statements on dog ownership on a -3 (big disadvantage) to + 3 (big advantage) scale. This unified approach allowed participants to weigh each item against others, which existing tools separating costs and benefits cannot do (e.g., as in^55^). Additionally, our method can identify which facets were perceived as positive by some owners, and as neutral or negative by others, something not possible with typical agreement Likert scales. Finally, we aimed to cover a wider range of costs and benefits than previous scales investigating the dog-owner relationship. For comparison, the MDORS scale focuses on the perceived emotional closeness between an owner and their dog, while overlooking the social and physical benefits of dog ownership.

Two open-ended questions were added to explore further, using qualitative techniques, what facets of dog ownership were perceived as the main advantages and disadvantages of having a dog. Using open-ended questions was also a way to potentially uncover important costs and benefits of dog ownership that were not mentioned in our list of items.

Defining the perceived costs and benefits of dog ownership in a comprehensive manner could raise awareness about the realities of sharing life with a dog. This knowledge could help reduce the gap between the expected positive outcomes of having a dog and the actual gains and costs of the relationship. This could ultimately enable prospective owners to engage in the dog adoption process in a more conscious and better-prepared way, benefiting both the humans and the dogs.

## Methods

### Ethics statement

The present study was part of a bigger project on the effects of dog ownership on human health. Participation was voluntary and anonymous. Respondents were informed of the main purpose of the study, and that their responses will be used for scientific purposes only. All of them gave their informed consent before starting the online questionnaire. Ethical approval was granted by the Hungarian United Ethical Review Committee for Research in Psychology. The ethical permission number is EPKEB 2023-91. All methods were carried out according to relevant guidelines and regulations for human participants.

### Data collection procedure

Participants were recruited online between May and November 2023 through social media platforms, mainly our Hungarian and international Dog Ethology and Family Dog Project Facebook pages and groups, which together have over 43,000 followers. Some users also shared our recruitment posts on their own Facebook or LinkedIn personal pages. Our survey was described to the participants as looking at the influence of dogs on human health and the reasons for having a dog or not. It was available in three languages (Hungarian, English, and French) and took about 35 min to complete. To prevent our results from being biased by cultural differences, the present study will focus on data collected using the Hungarian questionnaire only (N = 246).

There was no monetary compensation for participating in the study. However, Hungarian participants who completed the survey had the opportunity to enter a drawing to win one out of five 5000 HUF gift vouchers.

### Participants

Eligible criteria to be included in the present study were to be at least 18 years old and a dog owner. One participant was excluded because they answered for multiple dogs at the same time, and three others were excluded because they gave the same score (0 or 3) to all 33 items on the costs/benefits scale. Our sample was a convenience sample, in which the typical dog owner was a working woman in her forties who had received higher education and lived in a metropolitan area without financial difficulties. Likewise, the typical dog in our sample was about six years old, neutered and acquired as a young puppy. 67.5% of the respondents identified as the main caretaker of the dog. Table 1 summarises the characteristics of the respondents and their dogs.

**Table 1:** Owner and dog characteristics (N = 246).

Owner demographics		N	%
Gender	Women	228	92.7
	Men	18	7.3
Place of living	Metropolitan area	134	54.5
	Urban area	35	14.2
	Rural area	77	31.3
Financial status	No financial difficulties	60	24.4
	Getting by, with some budgeting	128	52
	Financial difficulties	58	23.6
Education level	Graduate school or lower	44	17.9
	Higher than graduate school	202	82.1
Working status	Working (full-time, part-time, self-employed)	213	86.6
	No work, other situations	33	13.4
Age (years)		Mean ± S.D: 41.1 ± 10.9	

Dog characteristics		N	%
Sex	Intact female	27	11
	Neutered female	107	43.5
	Neutered male	85	34.6
	Intact male	27	11
Source of acquisition	Breeder	100	40.7
	Rescue dog	108	43.9
	Other (from family/acquaintance, owner’s own breeding, online ad)	38	15.4
Age at acquisition	Before 12 weeks old	118	48
	Between 3 and 12 months old	65	26.4
	Adult (> 1 year old)	63	25.6
Purebred status	Purebred	142	57.7
	Mixed-breed	104	42.3
Age (years)		Mean ± S.D: 5.8 ± 3.8	
Dog’s primary caretaker	Respondent is the primary caretaker	166	67.5
	Care shared between the respondent and another person	80	32.5

### Instruments

In the following section, we will only describe the items that were used to measure the variables related to the main objective of the present study. Our questionnaire was initially developed in English. Some of its items (e.g., demographic questions) were selected from previous studies conducted in our department, which means they were already validated in both English and Hungarian. New items were translated into Hungarian following a committee approach. Two native speakers recruited at the ELTE university (i.e., the second author (BT) and an undergraduate student) independently translated the questionnaire into Hungarian and discussed the translations until they reached an agreement. The first version of the questionnaire was then piloted in each language on a small sample of volunteers in order to detect language mistakes, as well as unclear or equivocal items. The Hungarian translations were re-discussed by the translators when necessary (e.g., when a volunteer suggested a different wording). The final version of the English questionnaire can be found in the Supplementary material (Supplementary Material S1).

#### Owner and dog characteristics

We asked the participants about their (A) gender, (B) place of living, (C) financial status, (D) education level, (E) working status, and (F) age. Regarding their dog, the owners provided its (A) sex and neutering status, (B) source of acquisition, (C) age at acquisition, (D) breed, and (E) current age. We also asked who the dog’s primary caretaker was in the household. If an owner had more than one dog, they were asked to answer for the individual they felt the closest to.

#### Costs/benefits of dog ownership

We developed a scale (referred to as Cost/Benefit or ‘C/B’ scale) to investigate the perceived costs and benefits of dog ownership without a priori categorisation of costs and benefits of dog ownership, as some aspects can be perceived as either a burden or a benefit depending on the respondent’s circumstances and personal experience. To achieve this, we created 33 statements related to dog ownership, phrased as neutrally as possible using the conditional tense and general terms (e.g., *Dogs can cause harm to other animals*, *Having a dog can help people to learn responsibility*). Some of these statements were selected based on the most common costs and benefits described in previous literature^20,25,28,56,60,69,70^. Drawing on their expertise in the human–dog relationship, the authors decided together on additional statements relating to facets of dog ownership that have been less often addressed in quantitative studies. All items were rated on a scale from – 3 (big disadvantage) to + 3 (big advantage) and presented together in a single list. The order of the items was deliberately arranged by the authors so that items hypothesised to be perceived as positive were alternated with items expected to be viewed as more negative or ambiguous. Participants were explicitly instructed to rate each statement based on what it meant to them personally, rather than to society in general. If a statement did not apply to them or was neither a disadvantage nor an advantage in their view, they were asked to rate it as neutral (0). The original English phrasing and order of the 33 items are presented in the first two columns of Table 2.

**Table 2:** C/B scale descriptives (N = 246). The table indicates the mean score and standard deviation of each item, as well as its original phrasing, abbreviation and order. Items are ranked in descending order (from the most positive to the most negative item, thus, the higher mean score indicates higher perceived benefit (advantage) and the lower score higher perceived cost (disadvantage)).

Positively rated items (original phrasing)	Item order in the original questionnaire	Positively rated items (abbreviation)	Mean score ± SD
Dogs can brighten one’s life	23	Brighten life	2.78 ± 0.62
Having a dog can encourage people to be more physically active	4	Encourage physical activity	2.70 ± 0.68
Dogs can help their owner(s) to get through difficult situations in life	14	Help get through difficult life situations	2.59 ± 0.80
Dogs can be loyal and can provide unconditional love towards their owner(s)	31	Loyalty and unconditional love	2.58 ± 0.80
People can share fun moments of play and laughter with their dog	33	Play and laughter	2.55 ± 0.83
Dogs can give their owner(s) a sense of security and stability in life	29	Sense of security and stability in life	2.37 ± 0.98
Having a dog can help people to learn responsibility	9	Learn responsibility	2.36 ± 1.02
Dogs need the love and affection of their owner(s)	2	Need love and affection	2.23 ± 1.16
Having a dog can give people a reason to get up in the morning	7	Give a reason to get up in the morning	2.10 ± 1.20
Rescuing or adopting a dog can be a way to help another being in need, to contribute to a better world	26	Dog rescue contributes to a better world	2.05 ± 1.28
Having a dog can be a spiritual experience, and it could contribute to people’s self-growth	30	Spiritual experience and self-growth	2 ± 1.24
Dogs can contribute to a better family cohesion	20	Better family cohesion	2 ± 1.17
Having a dog can facilitate interactions with other people (e.g. during walks, training)	21	Facilitate interaction with others	1.99 ± 1.18
Dogs can keep children company	15	Keep children company	1.96 ± 1.33
Having a dog can give people the feeling of belonging to a community	8	Feeling to belong to a community	1.91 ± 1.26
Having a dog can have an influence on their owners’ daily routine	16	Influence daily routine	1.90 ± 1.39
Dogs need to be trained and educated	18	Need to be trained/educated	1.89 ± 1.26
Dogs need their owner(s) to take care of them	17	Need their owner to care for them	1.80 ± 1.30
Having a dog can have an influence on people’s quality of sleep	3	Influence sleep quality	1.63 ± 1.61
Time must be devoted to the dog on a daily basis	22	Time must be devoted to the dog	1.59 ± 1.54
Having a dog can be emotionally challenging (e.g. in case of illness, impairments that come with ageing, grief)	24	Emotional challenge	0.38 ± 2.05
*Average score of positively rated items*			2.06

Negatively rated items (original phrasing)	Item order in the original questionnaire	Negatively rated items (abbreviation)	Mean score ± SD
Dogs can cause allergies	1	Causing allergies	− 0.31 ± 0.79
Dogs can be noisy and barky	19	Noisy/barky	− 0.44 ± 1.19
Dogs can bring mess and dirt into the house	6	Mess/dirt into the house	− 0.53 ± 0.97
Dog owners can feel ashamed or uncomfortable because of the (problematic) behavior of their dog	27	Shame and discomfort due to the dog’s behaviour	− 0.53 ± 1.13
Dogs can damage the property of their owner(s)	32	Damage property	− 0.57 ± 1.05
The care of a dog can be expensive (e.g. food, vet bills, toys, brushes…)	10	Expensive care	− 0.61 ± 1.52
Having a dog can lead to conflicts with other people (non-owners, neighbors, etc.)	11	Conflicts with other people	− 0.61 ± 1.26
Dogs can cause harm to other animals	5	Harm other animals	− 0.63 ± 1.07
Dogs’ disobedience can generate feelings of frustration, stress, and anger	25	Anger and stress due to disobedience	− 0.64 ± 1.14
When on vacation, a solution to care for the dog may be needed	13	Vacation care	− 0.66 ± 1.41
Having a dog can make it difficult to find an appropriate place of living	12	Appropriate place of living	− 0.70 ± 1.35
Dogs usually have a shorter lifespan than their owner(s)	28	Short lifespan	− 1.67 ± 1.65
*Average score of negatively rated items*			− 0.66

In addition to this scale, we added two open-ended questions to capture costs and benefits that might not have been previously listed: ‘According to you, what is the biggest benefit of having a dog?’ and secondly, the same question where ‘benefit’ has been replaced by ‘cost’.

### Data analysis

#### Quantitative data

Statistical analysis was conducted using R.4.3.3 software. Principal Component Analysis (PCA) was conducted on the 33 items of the C/B scale. To determine the optimal number of components based on our data, we used a scree plot and parallel analysis (‘fa.parallel’ function of the ‘psych’ package^71^). We first standardised the data by centering and scaling it (‘scale’ function of the ‘base’ package^72^). PCA, conducted on a correlation matrix with an oblique rotation (oblimin), was run using the ‘principal’ function of the ‘psych’ package^71^. Due to the exploratory nature of the present work, we chose an oblique rotation because it allows components to correlate. A cut-off value of 0.40 was chosen in order to obtain components that were reliable enough, given our sample size (N = 246)^73^. Items that were not loading on any components were removed from the final model in a step-by-step manner. Cronbach’s alphas were calculated to determine the internal consistency of each extracted component. We also ran a Kaiser–Meyer–Olkin (KMO) test (‘KMO’ function of the ‘psych’ package^71^) and a Bartlett’s test of sphericity (‘cortest.bartlett’ function of the ‘psych’ package^71^) to ensure that our dataset was suitable for factor analysis. KMO index value was 0.88, exceeding the recommended value of 0.60^74^ and confirming that the correlations among variables were sufficient for factor analysis. All KMO values for individual items exceeded 0.70, indicating sufficient sampling adequacy for each item. Additionally, Bartlett’s test of sphericity was significant (χ^2^ (528) = 3306.9, *p* < 0.001), showing significant relationships among the variables. These results support that the sample was suitable for PCA.

#### Qualitative data

To analyse our qualitative data, we used an inductive, data-driven thematic analysis^75,76^. The authors first familiarised themselves with the corpus and took notes of their overall impressions of the data. Next, the first author (LG) manually attributed initial codes to each response, line-by-line. These initial codes were reviewed and refined until each code was clearly defined and distinct from others. Both semantic and latent meanings were coded, as the same costs and benefits could be expressed in different words by the owners. Themes were then created from the identified codes. They were not mutually exclusive, meaning that respondents could mention more than one theme in their answers. All authors discussed the codebook and agreed upon its final version. To determine how often these codes and themes appeared among the respondents, we coded their presence (1) or absence (0) in each response. We then calculated their frequency of appearance by summing the total occurrences and dividing by the number of respondents. We decided to retain low-frequency codes (i.e., representing 5% or less of the total dataset) in our descriptive analysis, as they were deemed to carry relevant, informative information for our topic. To assess inter-rater reliability, a second coder independently coded N = 99 benefit answers and N = 103 cost answers. Cohen’s Kappa coefficients were calculated in R using the ‘cohen.kappa’ function from the ‘psych’ package^71^. Low-frequency codes were excluded from this analysis. If Cohen’s Kappa coefficients fell below the 0.60 threshold, the code definitions were refined, and the coding updated (if necessary) until inter-rater agreement was deemed to be at least moderate (Cohen’s Kappa ≥ 0.60^77^).

Qualitative and quantitative data is available as a supplemental dataset.

## Results

### Quantitative analysis

#### Costs/Benefits of dog ownership scale: descriptive results

The five greatest perceived advantages of dog ownership were the following: *Dogs can brighten one’s life* (M = 2.78, SD = 0.62), *Having a dog can encourage people to be more physically active* (M = 2.70, SD = 0.68), *Dogs can help their owner(s) to get through difficult situations in life* (M = 2.59, SD = 0.80), *Dogs can be loyal and can provide unconditional love towards their owner(s)* (M = 2.58, SD = 0.80) and *People can share fun moments of play and laughter with their dog* (M = 2.55, SD = 0.83). The average rating of positively perceived statements was 2.06.

In contrast, the average rating of negatively perceived statements was −0.66, notably distant from the lower end of the scale (−3). Respondents perceived the following statements as the greatest disadvantages of having a dog: *Dogs usually have a shorter lifespan than their owner(s)* (M = −1.67, SD = 1.65), *Having a dog can make it difficult to find an appropriate place of living* (M = −0.70, SD = 1.35), *When on vacation, a solution to care for the dog may be needed* (M = -0.66, SD = 1.41), *Dogs’ disobedience can generate feelings of frustration, stress, and anger* (M = -0.64, SD = 1.14), and *Dogs can cause harm to other animals* (M = −0.63, SD = 1.07) (Table 2).

#### Principal component analysis

The point of inflexion on the scree plot clearly indicated that the best model contained three components, which was confirmed by the parallel analysis. Two items (*Dogs need the love and affection of their owner(s)* and *Dogs usually have a shorter lifespan than their owner(s)*) did not reach the 0.40 cut-off and were therefore excluded from the model (Table 3). One item loaded on two components above the cut-off value of 0.40: *When on vacation, a solution to care for the dog may be needed*. The first component (α = 0.90) included positive effects of dog keeping, both emotionally, physically and socially (e.g., laughter, love, family cohesion, feeling of belonging to a community, physical activity), and so it was labelled as *Emotional, physical and social benefits*. The second component (α = 0.85) focused on the negative emotions (e.g. shame, anger, grief) and practical challenges (e.g. housing, expensive care, damages) related to having a dog. We labelled it *Negative emotions and practical challenges*. The third component (α = 0.82) encompassed items related to facets of dog ownership which are neither clear benefits nor clear costs: the commitment and responsibilities involved in owning a dog. This component included day-to-day commitment (e.g., dedicating time, having a daily routine), the necessity to fulfil the dog’s needs (i.e., education and care) and the emotional challenges that come with living with a dog over time. We labelled this component *Commitment and responsibilities*. Together, these three components explained 42.7% of the total variance.

**Table 3:** Results of the Principal Component Analysis conducted on the *Perceived costs and benefits of dog ownership* scale (N = 246).

Perceived costs and benefits of dog ownership (N = 246)	Factor loading		
	Emotional, physical and social benefits	Negative emotions and practical challenges	Commitment and responsibilities
Spiritual experience and self-growth	**0.737**	0.092	− 0.252
Better family cohesion	**0.705**	− 0.016	0.035
Loyalty and unconditional love	**0.705**	0.042	− 0.080
Facilitate interaction with others	**0.686**	− 0.058	0.116
Sense of security and stability in life	**0.685**	0.019	0.114
Learn responsibility	**0.676**	− 0.118	0.160
Play and laughter	**0.636**	0.072	0.009
Help get through difficult life situations	**0.637**	− 0.116	0.179
Dog rescue contributes to a better world	**0.610**	0.117	− 0.239
Feeling to belong to a community	**0.580**	− 0.003	0.157
Brighten life	**0.574**	0.074	0.129
Encourage physical activity	**0.540**	− 0.071	0.203
Keep children company	**0.506**	− 0.039	0.045
Give a reason to get up in the morning	**0.470**	− 0.020	0.286
Harm other animals	0.078	**0.747**	− 0.138
Conflicts with other people	0.011	**0.698**	0.033
Damage property	− 0.022	**0.684**	− 0.013
Appropriate place of living	− 0.167	**0.681**	0.126
Shame and discomfort due to the dog’s behaviour	0.125	**0.604**	− 0.164
Mess/dirt into the house	0.078	**0.592**	0.065
Noisy/barky	0.001	**0.578**	0.233
Anger and stress due to disobedience	− 0.024	**0.569**	0.079
Expensive care	− 0.045	**0.541**	0.304
Causing allergies	0.098	**0.484**	− 0.202
Vacation care	− 0.152	**0.451**	**0.434**
Influence daily routine	0.084	-0.004	**0.726**
Time must be devoted to the dog	0.092	0.128	**0.698**
Need to be trained/educated	0.020	0.067	**0.670**
Need their owner to care for them	0.245	0.003	**0.642**
Influence sleep quality	0.168	− 0.034	**0.585**
Emotional challenge	0.062	0.214	**0.494**
Eigenvalue	5.75	4.22	3.29
% of variance	18.5	13.6	10.6
Cronbach α	0.90	0.85	0.82

Item loadings ≥ 0.40 are in bold.

### Qualitative analysis

#### Greatest benefits of dog ownership

**Fig. 1:**
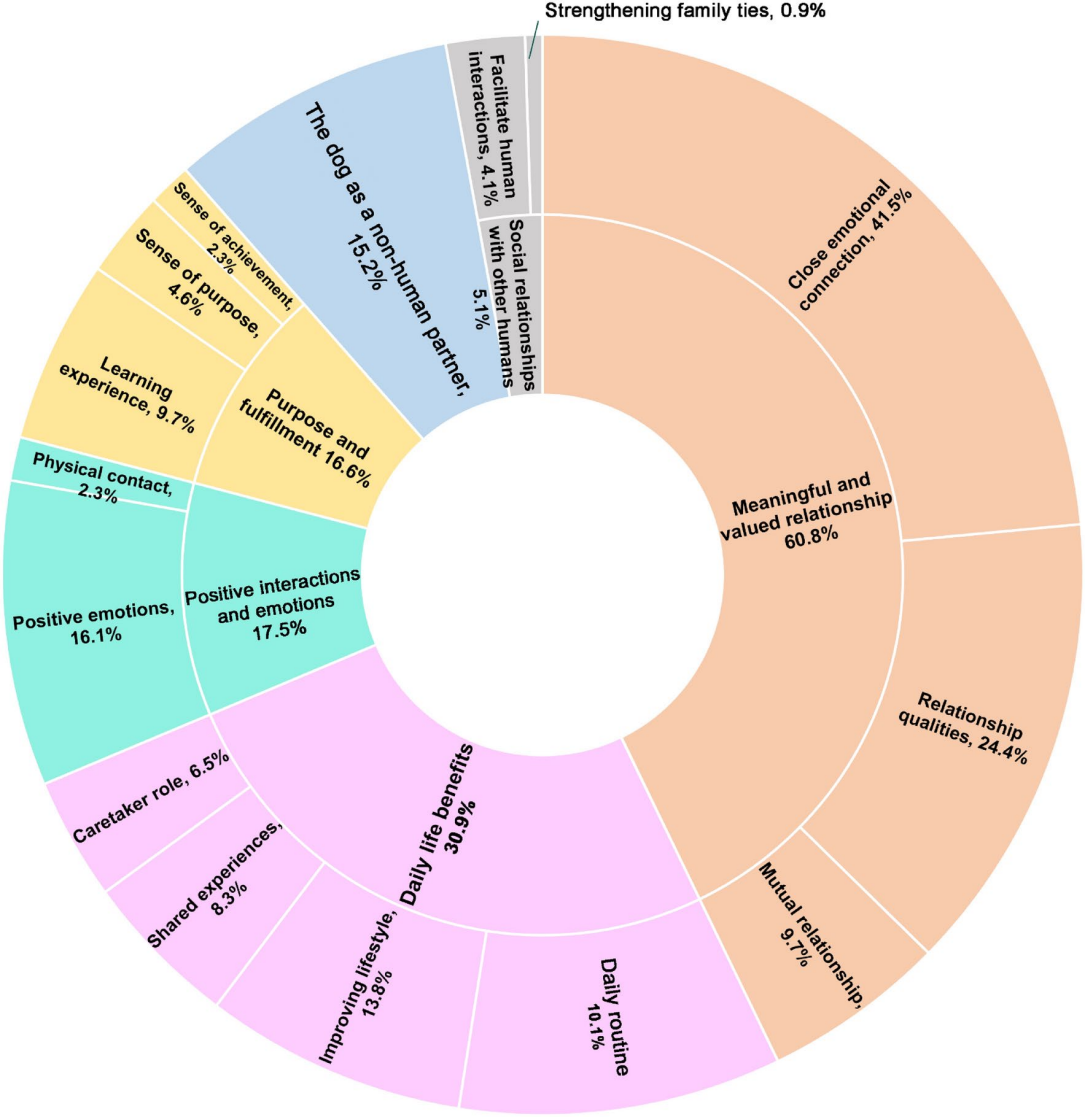
Distribution of the responses given to the ‘biggest benefit of dog ownership’ question (N = 217 respondents). The chart shows our six themes (inner ring) and their associated codes (outer ring). Percentages indicate how often each code and theme was mentioned by respondents. Respondents could provide multiple codes across different themes in their answers. For this reason, the sum of the segments in the pie chart exceeds 100%.

Out of the 246 participants in the study, 222 (90%) provided an answer to the following open-ended question: ‘According to you, what is the biggest benefit of having a dog?’. The answers of five respondents were not included in the coding because they were considered too vague (two respondents mentioned ‘everything’ and a ‘long list’, one simply answered the word ‘dog’, and two did not see life with a dog as having advantages or disadvantages). The remaining 217 respondents mentioned an average of 1.7 benefits. In total, we identified 15 codes distributed in six themes. One of the themes (The dog as non-human partner) did not divide into multiple codes. Figure 1 summarises the frequencies of occurrence of each benefit-related theme and code in the dataset. Table 4 shows the inter-rater reliability of each code (Cohen’s Kappa, N = 99).

**Table 4:** Inter-rater reliability was calculated using Cohen’s Kappa (N = 99). We did not check inter-rater reliability for codes with a frequency of 5% or less.

Benefit theme	Benefit code	Frequency (%)	Cohen’s Kappa coefficient
Meaningful and valued relationship	Close emotional connection	41.5	0.89
	Relationship qualities	24.4	0.80
	Mutual relationship	9.7	0.78
Daily life benefits	Improving lifestyle	13.8	0.78
	Daily routine	10.1	0.82
	Shared experiences	8.3	0.89
	Caretaker role	6.5	0.71
Positive interactions and emotions	Positive emotions	16.1	0.83
	Physical contact	2.3	Too low frequency for analysis
Purpose and fulfilment	Learning experience	9.7	0.68
	Sense of purpose	4.6	Too low frequency for analysis
	Sense of achievement	2.3	Too low frequency for analysis
The dog as non-human partner		15.2	0.91
Social relationships with other humans	Strengthening family ties	0.9	Too low frequency for analysis
	Facilitate human interactions	4.1	Too low frequency for analysis

##### Theme 1: a meaningful and valued relationship

The first theme described a meaningful and valued relationship. It was the predominant theme across the answers, with 60.8% of dog owners citing it. Its first code was cited in 41.5% of the responses and referred to a *close emotional connection* existing between the dog and its owner, with an emphasis on the love given by the dog. References to attachment and empathy were included as well:

"*The unconditional love that the dog makes you feel every day.*" (owner #154)

"*Experiencing, accepting, and learning pure love.*" (owner #126)

24.4% of the respondents also cited the many *relationship qualities* they saw in their dogs, the most important one being companionship. They referred to qualities such as selflessness, honesty, support, presence, acceptance, devotion, respect, and trust as well:

"*There is always someone around me, I’m not alone, there’s someone to talk to.*" (owner #185)

"*Faithful companion, always with us*." (owner #164)

Finally, 9.7% of the owners evoked the *reciprocity of the relationship*. They referred to a feeling of togetherness and belonging, and to the dog-owner communication. Expressions such as ‘giving and receiving love’ were counted, too. Finally, some owners mentioned the fact that this was a relationship they chose, similar to friendship:

"*Learning to be part of a team with the dog*." (owner #142)

"*Experiencing mutual development and a completely different level of relationship based on absolute honesty and reciprocity*." (owner #178)

##### Theme 2: daily life benefits

The second most frequent benefit was mentioned by 30.9% of the dog owners. It referred to four main aspects of sharing their daily lives with their dogs. First, dog ownership was perceived by 13.8% of the respondents as *improving daily lifestyle* by increasing activity and exercise levels, bringing energy and movement to life, and encouraging people to go out:

"*I spend much more time outdoors, in nature (with my dog)*." (owner #244)

"*A lot of extra energy they give*." (owner #47)

Another important aspect for 10.1% of the owners was the *daily routine* coming with the care of a dog. They also had the feeling that this routine structured their lives and provided them with stability and consistency:

"[...] *Due to the daily routine, they bring order into everyday life.* [...]" (owner #167)

"*A stable point, a steady presence in my life*." (owner #50)

The next code that belonged to the daily life theme was the *shared experiences* one. 8.3% of the owners highlighted the quality time spent with their dogs, and the activities they do together, including playing and walking:

"*Living through the shared moments with them*." (owner #49)

"[...] *we can do a thousand and one things together* [...]" (owner #3)

The last aspect of the daily life theme was the *caretaker role*. 6.5% of the respondents indicated that they liked being responsible for the dog, taking care of it, and teaching it:

"[...] *I enjoy taking responsibility for every task*." (owner #133)

##### Theme 3: positive interactions and emotions

The third theme relates to the positive interactions and emotions experienced with the dog. 17.5% of owners made references to it. *Positive emotions* described by 16.1% of the owners mostly encompassed happiness, laugh, but also relaxation, security, and comfort:

"*They make life more colourful*." (owner #51)

"*Even on the worst days, they can bring a smile to one’s face*." (owner #229)

"[...]* they make us laugh* [...]" (owner #13)

*Physical contact* was also highlighted by 2.3% of the owners:

"*No matter how the day went, it’s certain that we cuddle on the couch in the evening*." (owner #10)

##### Theme 4: purpose and fulfilment

This fourth theme was mentioned by 16.6% of the respondents. 9.7% of the owners described dog ownership as a *learning experience* about themselves, contributing to self-growth and self-awareness:

"*For me, the greatest advantage of having a dog is that I learn and grow a lot through my dog, I have become a much better person since I consciously try to be a ""good owner.""* [...]" (owner #30)

Dog ownership gave a *sense of purpose* to 4.6% of the owners, who perceived their lives as more enriched and full thanks to the dog. Additionally, the dog gave them a reason to live and generated motivation:

"[...] *the fact that I cannot give up. Ever*." (owner #210)

Finally, 2.3% of the owners mentioned a *sense of achievement* through dog ownership, whether it was because they felt they saved a dog’s life, they fulfilled their dreams or were proud to be able to take care of a dog:

"*Achieve dreams/plans that I always wanted to accomplish with a dog* [...]" (owner #232)

##### Theme 5: the dog as a non-human partner

15.2% of the respondents specifically highlighted the dog’s species as making the dog-owning experience special to them. They valued their special relationship with a non-human animal and appreciated the diverse qualities that dogs possess in their eyes:

"[...] *Being responsible for a creature not of my own species challenges me every day, which has taught me to see the world from a different perspective*. [...]" (owner #30)

"*Since I can’t mention a disadvantage, EVERYTHING that a dog can mean*." (owner #14)

"*Simply the fact that they exist*." (owner #6)

"*Dogs. Wonderful animals, funny, enthusiastic*." (owner #192)

##### Theme 6: social relationship with other humans

The least frequently mentioned benefit of dog ownership in our sample (5.1%) referred to how having a dog improved the social life of the owner, whether it was through *facilitating social interactions with other humans* (4.1% of the owners cited it) or *strengthening family ties* (0.9% of the owners cited it):

"[...] *a world opening to other human companions*." (owner #54)

"[...] *my adult children live in another city... most of our communication is centered around* [name of the dog]*... and this is good for them too, not just for me*." (owner #194)

#### Greatest costs of dog ownership

**Fig. 2:**
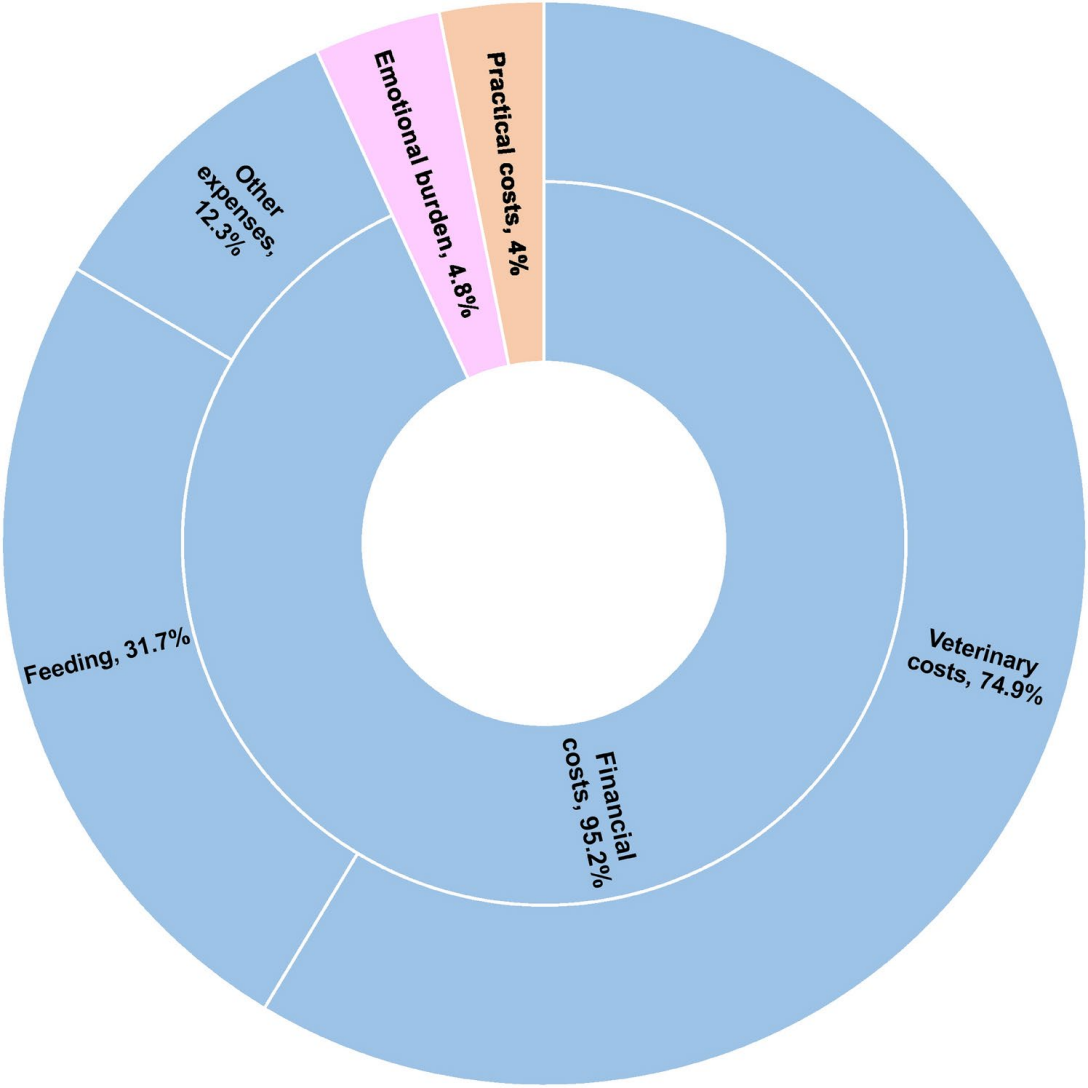
Distribution of the responses given to the ‘highest cost of dog ownership’ question (N = 225 respondents). Each inner ring represents one of the three themes, while outer rings represent the codes associated with the *Financial costs* theme and their frequencies (%). The two other themes were not subdivided into codes. A respondent could mention several responses belonging to more than one theme, therefore, the sum of the segments in the pie chart exceeds 100%.

Two hundred and thirty-two (94%) respondents answered the following open-ended question: ‘According to you, what is the biggest cost of having a dog?’. However, one response was excluded as we assumed it to be an answer to the previous question (i.e. the respondent mentioned unconditional love and respect), and two others because they were not specific enough (i.e., ‘it depends’, ‘the keeping itself’). Interestingly, two owners explicitly did not want to mention the costs of dog ownership (i.e., ‘you shouldn’t count it’, ‘it doesn’t matter’). The remaining 227 respondents gave an average of 1.3 costs. In total, three themes were identified; one of them (Theme 1) being composed of three distinct codes. Figure 2 summarises the frequencies of occurrence of each cost-related theme and code in the dataset, while Table 5 shows the inter-rater reliability of each code (Cohen’s Kappa, N = 103).

**Table 5:** Inter-rater reliability was calculated using Cohen’s Kappa (N = 103). We did not check inter-rater reliability for codes with a frequency of 5% or less.

Cost theme	Cost code	Frequency (%)	Cohen’s Kappa coefficient
Financial costs	Veterinary costs	74.9	0.98
	Feeding	31.7	0.96
	Other expenses	12.3	0.80
Emotional burden		4.8	Too low frequency for analysis
Practical costs		4	Too low frequency for analysis

##### Theme 1: financial costs

95.2% of the dog owners agreed that financial costs were the highest costs of dog ownership. More precisely, 74.9% of the respondents mentioned the *veterinary costs* relative to the expected and unexpected illnesses, treatments (e.g., medication, supplements, preventive treatment against worms), and surgeries:

"[...] *for us, for example, recognizing and treating a recently developed food allergy was a significant cost* [...]" (owner #111)

"*Veterinary costs, any surgery involving anesthesia is expensive, and if you have to take them to the vet during on-call hours, the on-call fee itself is also significant, unfortunately*." (owner #132)

The other main financial cost pointed out by 31.7% of the owners was *feeding* (e.g., daily feeding, special/high quality dog food):

"*Feeding, especially if it’s high-quality. But it pays off because the dog is healthier this way*." (owner #202)

"*Providing excellent quality food throughout the dog’s life*." (owner #224)

12.3% of the owners also mentioned *other expenses*, which included the financial cost of dog training, shared activities, equipment, grooming, and other unspecified general expenses:

"[...] *For me, the training (dog + owner) and the costs related to working together and sports are the highest.*" (owner #52)

"*Lifestyle sports, extra hobbies (foreign trips, etc.)*." (owner #71)

##### Theme 2: emotional burden

This second theme was mentioned by 4.8% of the owners. They referred to negative emotions arising due to unsolicited social interactions, anticipatory grief, and worry about health. Additionally, the emotional investment of dog ownership was perceived as the highest cost of dog ownership by one respondent:

"*Emotional and time investment, sacrificing comfort* [...]" (owner #100)

"*The pain when we lose them. My dog is still young, but I already fear the thought of having to say goodbye to each other someday*." (owner #190)

##### Theme 3: practical costs

Lastly, 4% of the respondents pointed out the practical costs of having a dog, including the limitations in activities and travelling, as well as the time that has to be dedicated to the dog (e.g. for care, socialisation of the dog):

"*I can’t take my dog everywhere with me*." (owner #26)

"*Time management and adaptation*." (owner #74)

## Discussion

Our study aimed to provide a comprehensive approach to the costs and benefits of dog ownership, as perceived by a convenience sample of dog owners assumed to be generally satisfied with their relationship with their dogs. We wanted to determine how the various facets of dog ownership were evaluated by this specific group of dog owners. To do so, we created a Cost/Benefit scale encompassing 33 neutrally-phrased statements about dog ownership that could be rated from being a great advantage through being neutral to being a great cost. This quantitative measure was complemented by the analysis of qualitative data collected on the same sample.

The first main finding of our study is that the best model to explain how owners perceive the different facets of dog ownership was more complex than a binary cost–benefit structure. Indeed, PCA revealed three main components, respectively referring to (1) the emotional, physical and social benefits of dog ownership, (2) the negative emotions and practical challenges of dog ownership, (3) the commitment and responsibilities coming with dog ownership. While the first two aspects can be paralleled with what is usually described in the literature as the costs and benefits of dog ownership (e.g.,^54,78^), the third aspect supports the hypothesis that not all owners find the same (dis)advantages in having a dog. More specifically, in our study, responsibility and commitment seemed to be the facets of dog ownership that divided owners the most. Items related, for instance, to the time and care that should be devoted to the dog, the influence of dog ownership on sleep quality and daily routine, and the emotional challenge of having an ill/old dog were rated positively by many owners and rated negatively or neutrally by many others. Likewise, the larger standard deviations of these items suggest more variability in responses among dog owners. These findings can be likened to those previously reported by Barcelos and colleagues^25,79^, who showed that some activities related to dog ownership, such as dog training and ownership routines, could be both positively and negatively valenced depending on context.

One item cross-loaded on two components: the need to find a solution for dog care during vacations. This item was perceived as both a practical challenge and a responsibility of dog ownership. Dog owners travelling abroad by public transportation (e.g., train, plane) and staying at hotels (which are not always pet-friendly) might encounter this practical issue more often than those travelling by car to a nearby relative or dog owners who do not take holidays at all. This challenge might also arise due to a lack of dedicated solutions for owners (e.g., limited and/or costly dog daycare services). At the same time, animal welfare organisations’ campaigns regularly insist on the necessity to keep in mind the long-term commitments that come with pet ownership, including during holiday time, when the pet might become a bigger burden (e.g.,^80^). However, it should be noted that seasonal effects on dog relinquishment are not systematically observed^81^.

Even in a convenience sample, it is likely that the dogs’ and owners’ characteristics vary enough to explain why the same facets of dog ownership can be experienced differently. For instance, the emotional challenge of caring for an aged/ill dog may not have been experienced by all respondents, depending on their previous ownership experiences and the health condition of their current dog (which we didn’t ask about in the current study). Some owners may also consider that the ageing of the dog is ‘part of life’ and, therefore, not a challenge, while others may find it much more difficult to accept. Qualitative data confirm this hypothesis as well. For example, while some owners declared enjoying the daily responsibilities and the routine coming with the care of a dog the most, others considered them to be among the highest costs of dog ownership.

These variations in how dog owners perceive the responsibility and commitment coming with dog ownership might also be one of the potential explanations for the absence of a generalised pet effect^13,79^. First, owner responsibilities can vary according to the dog’s needs. Owners of sick dogs or dogs with problematic behaviours are more likely to experience a greater caregiver burden than owners of healthy dogs, which is in turn associated with increased stress, poorer psycho-social functioning and lower well-being^22–24,82^. Some personal characteristics of the owner might also influence the perception of the caregiver role. For example, the more attachment avoidant an owner is (measured by psychological scales), the more they might be uncomfortable with the long-term commitment coming with dog ownership, and the less they might be involved in the caregiving of the dog^83,84^. Likewise, for some people, the inherent responsibilities of the dog-owner relationship can be perceived as more burdensome than those in human-to-human relationships. Indeed, the owner bears sole responsibility for the relationship with their dog, while in relationships between human adults, both parties are expected to share responsibility equally.

On the other hand, both quantitative and qualitative results indicate that some facets of dog ownership are almost unanimously perceived as positive or negative. The emotional, physical and social benefits of dog ownership (e.g., dogs brighten life, provide unconditional love to their owners and support them during difficult times) were rated the most positively, while the qualitative analysis revealed that the most frequently mentioned benefits were by far the close emotional connection existing between the dog and the owner, as well as the relationship qualities of the dog. Dog ownership was also seen as a meaningful learning experience that contributes to self-growth, similar to what was found in previous qualitative studies and reviews^20,45,61,79^. A few participants also pointed out that their relationship with the dog as a non-human species and physical contact with the dog were the greatest benefits of dog ownership, which were not included in our quantitative scale. These answers align with the biophilia hypothesis, which posits that humans are genetically predisposed to be attracted to and form (affective) bonds with natural elements, especially animals^85^. The biophilia effects of pet ownership could explain why some people benefit from their pets’ presence and physical interactions^79,86^.

In other words, many owners perceived dogs as genuine beings providing unconditional love and constant presence. Interestingly, most of these benefits can be paralleled with the answers given by parents of children^87,88^.

Conversely, like parenthood, dog ownership comes with its challenges. The majority of dog owners from our sample highlighted the financial costs of dog ownership in their open-text responses, especially in regard to health-related expenses. When they become too serious, financial challenges of pet ownership can lead to broader negative outcomes, and turn into an important psychological burden^42^. However, the expensive care of a dog was not among the C/B scale items that were the most negatively perceived. Similar to what was found by Barcelos et al.^25^, the most negative outcome of dog ownership was related to the dog’s short lifespan. Yet, the large standard deviation of this item suggests variations in how owners perceived this statement. Again, owners might differ in how much they accept the fact that their dog will most likely have a shorter lifespan than their own. Additionally, under some circumstances, the fact that dogs usually have shorter lifespans than their owners might be seen as a ‘good thing’. For instance, very old owners do not always plan ahead for what will happen to their dogs in case they predecease them^89,90^.

Another important finding was that, as predicted by the social exchange theory, the mean of the positively rated items of our quantitative scale was higher than the mean of the negatively rated items. This was confirmed in the qualitative analysis: on average, respondents mentioned a higher number of benefits than costs. Although this result was predictable, as typical dog owners, especially from a volunteer sample, would be expected to keep their dog because they benefit from it more than it costs them, it might not always mean that owners experiencing more costs than benefits would relinquish their dog right away. Indeed, despite having to deal with daily challenges such as canine behaviour problems or financial difficulties, the social pressure of being a ‘good owner’ might encourage some owners to maintain their relationship with their dogs at all costs. Judgment of others can generate feelings of shame and guilt in owners struggling with their dogs^91,92^, making it harder for them to terminate the relationship and relinquish the dog, especially when they love the dog and are concerned about its future.

The higher absolute mean rating of positive items compared to negative items might alternatively be explained by our use of self-reported measures. Indeed, even if we phrased our statements as neutrally as possible and anonymised data collection to reduce this issue, we acknowledge that self-reported data can be subject to several response biases, including social desirability bias^93^. Owners may have been prompted to present themselves as caring, responsible owners to conform to social norms. Another bias often discussed in pet-owner relationship studies is the pet-enhancement bias^94^, according to which people tend to present their pets in the best possible light to others, over-reporting their positive qualities. For all these reasons, some dog owners may have been reluctant to admit that their beloved companions could generate negative emotions, negatively impact their quality of life, or they could be messy, noisy and destructive.

Our findings are also limited by the sample surveyed. It is likely that our respondents, most of whom were recruited through our social media pages, had a particular interest in dog behaviour and understanding the dog–human relationship. Thus, they might have been more informed about dogs and more aware of the realities of dog ownership than the general population of dog owners. Their potential good preparation for life with a dog could also explain why they reported few costs associated with dog ownership. All in all, we acknowledge that surveying a convenience sample limits the generalisation of our findings, which describe a specific subgroup of dog owners who are not fully representative of most owners.

## Conclusion

Our comprehensive approach, using neutrally-phrased items, appears to be a reliable solution to quantify how negatively, positively or neutrally each facet of dog ownership is perceived. We found that the different facets of the dog–human relationship could be divided into three main components, going beyond a binary cost-and-benefit approach. While the emotional, physical, and social benefits and the negative emotions and practical challenges of dog ownership seemed to be homogeneously seen as positive or negative, there was greater variation in how the commitment and responsibility of owning a dog were regarded by our sample. Additionally, although our results suggest that people do indeed maintain their relationships with their dogs because they derive more benefits than costs, the next step would be to identify owners for whom this ratio is reversed. Therefore, future studies must take into account the human and dog factors that could potentially explain the variability in perception of the different facets of dog ownership. Identifying these factors would be of great social relevance by raising awareness about what positive and negative outcomes can be expected in regard to the prospective owner’s characteristics. This would also contribute to the literature aiming to describe how to make the best dog-owner match. Likewise, less easily reachable populations of dog owners (e.g., dissatisfied owners) should be included in future work. Finally, another crucial question regarding pet effect studies that was raised by our findings relates to what is measured as a positive effect of dog ownership and how it is measured. Indeed, most benefits cited by our respondents might be less easily quantifiable (e.g. self-growth, mere existence of the dog, love) than mental and physical health outcomes. Moreover, in some circumstances, it might be more (emotionally) costly for an owner to relinquish their dog despite the little benefit gained from the relationship, highlighting once again the necessity to investigate the potential factors mediating the link between dog ownership and human health.

## Supplementary Information

Supplementary Information 1.

Supplementary Information 2.

## Data Availability

The datasets generated and analysed during the current study are available as supplementary materials.

## References

[1] Kim, J. How COVID-19 Affected Pet Ownership in the US (Statistics to Know in 2024). *PangoVet* https://pangovet.com/pet-lifestyle/general/how-has-covid-affected-pet-ownership-us/ (2022).

[2] FEDIAF. Annual report 2023. *FEDIAF EuropeanPetFood*. https://europeanpetfood.org/about/annual-report/ (2023).

[3] Shahbandeh, M. Shahbandeh, M. Shahbandeh, Research expert covering agriculture & FMCG, & Shahbandeh. Number of pet owning households in the United States 2023. *Statista*. https://www.statista.com/statistics/198095/pets-in-the-united-states-by-type-in-2008/ (2024).

[4] Holland, K. E., Mead, R., Casey, R. A., Upjohn, M. M. & Christley, R. M. Why do people want dogs? A mixed-methods study of motivations for dog acquisition in the United Kingdom. *Front. Vet. Sci.* **9**, (2022). 10.3389/fvets.2022.877950 PMC9127952 35619602

[5] Powell, L. et al. Expectations for dog ownership: Perceived physical, mental and psychosocial health consequences among prospective adopters. *PLoS ONE* **13**, e0200276 (2018). 29979749 10.1371/journal.pone.0200276 PMC6034856

[6] Anderson, K. L., Holland, K. E., Casey, R. A., Cooper, B. & Christley, R. M. Owner expectations and surprises of dog ownership experiences in the United Kingdom. *Front. Vet. Sci.* **11**, (2024). 10.3389/fvets.2024.1331793 PMC10880448 38384957

[7] Herzog, H. Is Time With Pets Good for Our Cardiovascular Health? *Psychology Today* https://www.psychologytoday.com/us/blog/animals-and-us/202208/is-time-pets-good-our-cardiovascular-health (2022).

[8] Serpell, J., McCune, S., Gee, N. & Griffin, J. A. Current challenges to research on animal-assisted interventions. *Appl. Dev. Sci.* **21**, 223–233 (2017).

[9] Barcelos, A. M. et al. Dog owner mental health is associated with dog behavioural problems, dog care and dog-facilitated social interaction: A prospective cohort study. *Sci. Rep.* **13**, 21734 (2023). 38066034 10.1038/s41598-023-48731-z PMC10709316

[10] Bennett, B., Cosh, S., Thepsourinthone, J. & Lykins, A. A Mixed-Methods Assessment of Human Well-Being Related to the Presence of Companion Animals During the COVID-19 Pandemic. **5**, (2022).

[11] Branson, S., Boss, L., Cron, S. & Kang, D.-H. Examining differences between homebound older adult pet owners and non-pet owners in depression, systemic inflammation, and executive function. *Anthrozoös* **29**, 323–334 (2016).

[12] Flegr, J. & Preiss, M. Friends with malefit. The effects of keeping dogs and cats, sustaining animal-related injuries and Toxoplasma infection on health and quality of life. *PLOS ONE* **14**, 1988 (2019). 10.1371/journal.pone.0221988 PMC6874301 31756184

[13] Herzog, H. The impact of pets on human health and psychological well-being: Fact, fiction, or hypothesis?. *Curr. Dir. Psychol. Sci* **20**, 236–239 (2011).

[14] Amiot, C., Bastian, B. & Martens, P. People and companion animals: It takes two to tango. *BioScience* **66**, 552–560 (2016).

[15] Friedman, E. & Krause-Parello, C. A. Companion animals and human health: benefits, challenges, and the road ahead for human–animal interaction. *Rev. Sci. Tech. OIE* **37**, 71–82 (2018). 10.20506/rst.37.1.2741 30209428

[16] Gee, N. R. & Mueller, M. K. A systematic review of research on pet ownership and animal interactions among older adults. *Anthrozoös* **32**, 183–207 (2019).

[17] Islam, A. & Towell, T. Cat and dog companionship and well-being: A systematic review. *Int. J. Appl. Psychol.* **3**, 49–155 (2013).

[18] Martins, C. F. *et al.* Pet’s influence on humans’ daily physical activity and mental health: A meta-analysis. *Front. Public Health* **11**, (2023). 10.3389/fpubh.2023.1196199 PMC10262044 37325330

[19] Matchock, R. L. Pet ownership and physical health. *Current Opin. Psychiatry* **28**, 386 (2015). 10.1097/YCO.0000000000000183 26164613

[20] Merkouri, A., Graham, T. M., O’Haire, M. E., Purewal, R. & Westgarth, C. Dogs and the good life: A cross-sectional study of the association between the dog–owner relationship and owner mental wellbeing. *Front. Psychol.* **13**, 903647 (2022). 35923726 10.3389/fpsyg.2022.903647 PMC9341998

[21] Kogan, L. R., Bussolari, C., Currin-McCulloch, J., Packman, W. & Erdman, P. Dog owners: Disenfranchised guilt and related depression and anxiety. *Human-Animal Interact.* **2023**, 0016 (2023).

[22] Spitznagel, M. B., Jacobson, D. M., Cox, M. D. & Carlson, M. D. Caregiver burden in owners of a sick companion animal: A cross-sectional observational study. *Veter. Record* **181**, 321–321 (2017). 10.1136/vr.104295 28870976

[23] Spitznagel, M. B., Cox, M. D., Jacobson, D. M., Albers, A. L. & Carlson, M. D. Assessment of caregiver burden and associations with psychosocial function, veterinary service use, and factors related to treatment plan adherence among owners of dogs and cats. *J. Am. Vet. Med. Assoc.* **254**, 124–132 (2019). 30668290 10.2460/javma.254.1.124

[24] Christiansen, S. B., Kristensen, A. T., Sandøe, P. & Lassen, J. Looking after chronically III dogs: Impacts on the Caregiver’s life. *Anthrozoös* **26**, 519–533 (2013).

[25] Barcelos, A. M., Kargas, N., Maltby, J., Hall, S. & Mills, D. S. A framework for understanding how activities associated with dog ownership relate to human well-being. *Sci. Rep.* **10**, 11363 (2020). 32647301 10.1038/s41598-020-68446-9 PMC7347561

[26] Applebaum, J. W., Tomlinson, C. A., Matijczak, A., McDonald, S. E. & Zsembik, B. A. The concerns, difficulties, and stressors of caring for pets during COVID-19: Results from a large survey of U.S. Pet owners. *Animals* **10**, 1882 (2020). 33076475 10.3390/ani10101882 PMC7602525

[27] Chur-Hansen, A., Winefield, H. & Beckwith, M. Reasons given by elderly men and women for not owning a pet, and the implications for clinical practice and research. *J. Health Psychol.* **13**, 988–995 (2008). 18987070 10.1177/1359105308097961

[28] Fifield, S. J. & Forsyth, D. K. A pet for the children: Factors related to family pet ownership. *Anthrozoös* **12**, 24–32 (1999).

[29] Królaczyk, K., Kavetska, K. M. & Flis, K. Owning a samoyed dog—cost analysis. *Acta Scientiarum Polonorum Zootechnica* **20**, 71–76 (2021).

[30] Matijczak, A., Tomlinson, C. A., Applebaum, J. W., Kogan, L. R. & McDonald, S. E. Development and validation of the pet-related stress scale. *Pets* **1**, 70–87 (2024).

[31] Damborg, P. et al. Bacterial zoonoses transmitted by household pets: State-of-the-art and future perspectives for targeted research and policy actions. *J. Comp. Pathol.* **155**, S27–S40 (2016). 25958184 10.1016/j.jcpa.2015.03.004

[32] Duncan-Sutherland, N., Lissaman, A. C., Shepherd, M. & Kool, B. Systematic review of dog bite prevention strategies. *Inj Prev.* **28**, 288–297 (2022). 35393286 10.1136/injuryprev-2021-044477

[33] Martens, P., Su, B. & Deblomme, S. The ecological paw print of companion dogs and cats. *BioScience* **69**, 467–474 (2019). 31190686 10.1093/biosci/biz044 PMC6551214

[34] Morgan, G. et al. An investigation of the presence and antimicrobial susceptibility of enterobacteriaceae in raw and cooked kibble diets for dogs in the United Kingdom. *Front. Microbiol.* **14**, 1301841 (2023). 38260907 10.3389/fmicb.2023.1301841 PMC10800874

[35] Emerson, R. M. Social Exchange Theory. *Annual*. *Rev. Sociol.* **2**, 335–362 (1976).

[36] Netting, F. E., Wilson, C. C. & New, J. C. The human-animal bond: Implications for practice. *Soc. Work* **32**, 60–64 (1987).

[37] Junça-Silva, A. Friends with benefits: The positive consequences of pet-friendly practices for workers’ well-being. *Int. J. Environ. Res. Public Health* **19**, 1069 (2022). 35162092 10.3390/ijerph19031069 PMC8834589

[38] Leslie, B. E., Meek, A. H., Kawash, G. F. & McKeown, D. B. An epidemiological investigation of pet ownership in Ontario. *Can Vet. J.* **35**, 218–222 (1994). 8076276 PMC1686751

[39] Westgarth, C. et al. Factors associated with dog ownership and contact with dogs in a UK community. *BMC Veter. Res.* **3**, 5 (2007). 10.1186/1746-6148-3-5 PMC1852100 17407583

[40] Endenburg, N., Hart, H. T. & Bouw, J. Motives for acquiring companion animals. *J. Econ. Psychol.* **15**, 191–206 (1994).

[41] Lambert, K., Coe, J., Niel, L., Dewey, C. & Sargeant, J. M. A systematic review and meta-analysis of the proportion of dogs surrendered for dog-related and owner-related reasons. *Prev. Vet. Med.* **118**, 148–160 (2015). 25466216 10.1016/j.prevetmed.2014.11.002

[42] Muldoon, J. C. & Williams, J. M. When having a pet becomes a luxury you can no longer afford. *Anthrozoös* **37**, 881–904 (2024).

[43] New, J. C. Jr. et al. Characteristics of shelter-relinquished animals and their owners compared with animals and their owners in U.S. pet-owning households. *J. Appl. Anim. Welfare Sci.* **3**, 179–201 (2000).

[44] O’Connor, R., Coe, J. B., Niel, L. & Jones-Bitton, A. Effect of adopters’ lifestyles and animal-care knowledge on their expectations prior to companion-animal guardianship. *J. Appl. Anim. Welfare Sci.* **19**, 157–170 (2016). 10.1080/10888705.2015.1125295 26865430

[45] Maharaj, N. & Haney, C. J. A qualitative investigation of the significance of companion dogs. *West J. Nurs. Res.* **37**, 1175–1193 (2015). 25092206 10.1177/0193945914545176

[46] McNicholas, J. et al. Pet ownership and human health: A brief review of evidence and issues. *BMJ* **331**, 1252–1254 (2005). 16308387 10.1136/bmj.331.7527.1252 PMC1289326

[47] Sutcliffe, A., Dunbar, R., Binder, J. & Arrow, H. Relationships and the social brain: Integrating psychological and evolutionary perspectives. *Br. J. Psychol.* **103**, 149–168 (2012). 22506741 10.1111/j.2044-8295.2011.02061.x

[48] Purewal, R. et al. Companion animals and child/adolescent development: A systematic review of the evidence. *Int. J. Environ. Res. Public Health* **14**, 234 (2017). 28264460 10.3390/ijerph14030234 PMC5369070

[49] Crowell-Davis, S. L. Motivation for pet ownership and its relevance to behavior problems. *Compend. Contin. Educ. Vet.* **30**, 423-4, 427-8 (2008). 19086251

[50] Dattani, S., Rodés-Guirao, L., Ritchie, H. & Roser, M. Mental health. *Our World in Data*. https://ourworldindata.org/mental-health (2023).

[51] Ellis, A., Stanton, S. C. E., Hawkins, R. D. & Loughnan, S. The link between the nature of the human-companion animal relationship and well-being outcomes in companion animal owners. *Animals* **14**, 441 (2024). 38338084 10.3390/ani14030441 PMC10854534

[52] Garrity, T. F., Stallones, L. F., Marx, M. B. & Johnson, T. P. Pet ownership and attachment as supportive factors in the health of the elderly. *Anthrozoös* **3**, 35–44 (1989).

[53] GonzálezRamírez, M. T. & LanderoHernández, R. Benefits of dog ownership: Comparative study of equivalent samples. *J. Veter. Behav.* **9**, 311–315 (2014).

[54] Hawkins, R. D., Hawkins, E. L. & Tip, L. “I can’t give up when i have them to care for”: People’s experiences of pets and their mental health. *Anthrozoös* **34**, 543–562 (2021).

[55] Bouma, E. M. C., Vink, L. M. & Dijkstra, A. Expectations versus reality: Long-term research on the dog-owner relationship. *Animals* **10**, 772 (2020). 32365588 10.3390/ani10050772 PMC7278369

[56] Dwyer, F., Bennett, P. C. & Coleman, G. J. Development of the monash dog owner relationship scale (MDORS). *Anthrozoös* **19**, 243–256 (2006).

[57] Meyer, I. & Forkman, B. Dog and owner characteristics affecting the dog–owner relationship. *J. Veter. Behav.* **9**, 143–150 (2014).

[58] van Herwijnen, I. R., van der Borg, J. A. M., Naguib, M. & Beerda, B. Dog ownership satisfaction determinants in the owner-dog relationship and the dog’s behaviour. *PLOS ONE* **13**, e0204592 (2018). 30235347 10.1371/journal.pone.0204592 PMC6147508

[59] Tversky, A. & Kahneman, D. The framing of decisions and the psychology of choice. *Science* **211**, 453–458 (1981). 7455683 10.1126/science.7455683

[60] Bryant, B. K. The richness of the child-pet relationship: A consideration of both benefits and costs of pets to children. *Anthrozoös* **3**, 253–261 (1990).

[61] Cyr, K. & Hawkins, R. D. Pets and prams: Exploring perceptions of companion animals in relation to maternal wellbeing. *Anthrozoös* **37**, 745–763 (2024).

[62] Meier, C. & Maurer, J. Buddy or burden? Patterns, perceptions, and experiences of pet ownership among older adults in Switzerland. *Eur. J. Ageing* **19**, 1201–1212 (2022). 36506656 10.1007/s10433-022-00696-0 PMC9729639

[63] Zoanetti, J., Young, J. & Nielsen, T. D. A scoping review of the risks posed by companion animals to older adults. *Anthrozoös* **37**, 1015–1031 (2024).

[64] Hayden-Evans, M., Milbourn, B. & Netto, J. ‘Pets provide meaning and purpose’: A qualitative study of pet ownership from the perspectives of people diagnosed with borderline personality disorder. *Adv. Mental Health* **16**, 152–162 (2018).

[65] Packer, R. M. A., O’Neill, D. G., Fletcher, F. & Farnworth, M. J. Great expectations, inconvenient truths, and the paradoxes of the dog-owner relationship for owners of brachycephalic dogs. *PLOS ONE* **14**, e0219918 (2019). 31323057 10.1371/journal.pone.0219918 PMC6641206

[66] González-Ramírez, M. T., Vanegas-Farfano, M. & Landero-Hernández, R. Differences in stress and happiness between owners who perceive their dogs as well behaved or poorly behaved when they are left alone. *J. Veter. Behav.* **28**, 1–5 (2018).

[67] Kuntz, K., Ballantyne, K. C., Cousins, E. & Spitznagel, M. B. Assessment of caregiver burden in owners of dogs with behavioral problems and factors related to its presence. *J. Veter. Behav.* **64–65**, 41–46 (2023).

[68] Amiot, C., Gagné, C. & Bastian, B. Pet ownership and psychological well-being during the COVID-19 pandemic. *Sci. Rep.* **12**, 6091 (2022). 35413973 10.1038/s41598-022-10019-z PMC9002031

[69] Crawford, E. K., Worsham, N. L. & Swinehart, E. R. Benefits derived from companion animals, and the use of the term “attachment”. *Anthrozoös* **19**, 98–112 (2006).

[70] González-Ramírez, M. T., Landero-Hernández, R. & Vanegas-Farfano, M. The effects of dog-owner relationship on perceived stress and happiness. *Human-Animal Interact. Bull.* **2018**, (2018).

[71] Revelle, W. psych: Procedures for Psychological, Psychometric, and Personality Research (R package version 2.3.6. https://www.rdocumentation.org/packages/psych/versions/2.3.6 (2023).

[72] R Core Team. base-package: The R Base Package. https://rdrr.io/r/base/base-package.html (2024).

[73] Stevens, J. P. *Applied Multivariate Statistics for the Social Sciences, 5th Ed*. xii, 651 (Routledge/Taylor & Francis Group, New York, NY, US, 2009).

[74] Kaiser, H. F. An index of factorial simplicity. *Psychometrika* **39**, 31–36 (1974).

[75] Braun, V. & Clarke, V. Using thematic analysis in psychology. *Qual. Res. Psychol.* **3**, 77–101 (2006).

[76] Byrne, D. A worked example of Braun and Clarke’s approach to reflexive thematic analysis. *Qual Quant* **56**, 1391–1412 (2022).

[77] McHugh, M. L. Interrater reliability: The kappa statistic. *Biochem. Med. (Zagreb)* **22**, 276–282 (2012). 23092060 PMC3900052

[78] Wood, L. et al. The pet factor—companion animals as a conduit for getting to know people, friendship formation and social support. *PLOS ONE* **10**, e0122085 (2015). 25924013 10.1371/journal.pone.0122085 PMC4414420

[79] Barcelos, A. M., Kargas, N., Maltby, J. & Mills, D. S. Potential psychosocial explanations for the impact of pet ownership on human well-being: Evaluating and expanding current hypotheses. *Human-Animal Interactions* **2023**, (2023).

[80] Société Protectrice des Animaux. ‘Il était une fois… L’Abandon’ : Nouveau film de sensibilisation de la SPA. https://www.la-spa.fr/articles/il-etait-une-fois-l-abandon/ (2023).

[81] Fatjó, J. et al. Epidemiology of dog and cat abandonment in Spain (2008–2013). *Animals* **5**, 426–441 (2015). 26479243 10.3390/ani5020364 PMC4494419

[82] Britton, K. et al. Caregiving for a companion animal compared to a family member: Burden and positive experiences in caregivers. *Front. Vet. Sci.* **5**, 325 (2018). 10.3389/fvets.2018.00325 PMC6308119 30619903

[83] Coy, A. E. & Green, J. D. Treating pets well: The role of attachment anxiety and avoidance. *Human-animal interaction bulletin* **2018**, (2018).

[84] Lockyer, J. M. & Oliva, J. L. Better to have loved and lost? human avoidant attachment style towards dogs predicts group membership as ‘forever owner’ or ‘foster carer’. *Animals* **10**, 1679 (2020). 32957574 10.3390/ani10091679 PMC7552168

[85] Wilson, E. O. *Biophilia*. (Harvard University Press, 1984).

[86] Delanoeije, J. & Verbruggen, M. Biophilia in the home–workplace: Integrating dog caregiving and outdoor access to explain teleworkers’ daily physical activity, loneliness, and job performance. *J. Occup. Health Psychol.* **29**, 131–154 (2024). 38913702 10.1037/ocp0000378

[87] Nelson, S. K., Kushlev, K. & Lyubomirsky, S. The pains and pleasures of parenting: When, why, and how is parenthood associated with more or less well-being?. *Psychol. Bull.* **140**, 846–895 (2014). 24491021 10.1037/a0035444

[88] Nomaguchi, K. & Milkie, M. A. Parenthood and well-being: A decade in review. *J. Marriage Fam.* **82**, 198–223 (2020). 32606480 10.1111/jomf.12646 PMC7326370

[89] Beyer, G. W. & Seltzer, B. Don’t forget about pets when planning for disability and death. *Gener. J. Am. Soc. Aging* **42**, 109–112 (2018).

[90] Smith, D. W. E., Seibert, C. S., Jackson, F. W. & Snell, J. Pet ownership by elderly people: Two new issues. *Int. J. Aging Hum. Dev.* **34**, 175–184 (1992). 1582711 10.2190/DQUW-XAE9-8CYH-F04J

[91] Buller, K. & Ballantyne, K. C. Living with and loving a pet with behavioral problems: Pet owners’ experiences. *J. Veter. Behav.* **37**, 41–47 (2020).

[92] Kogan, L. R., Bussolari, C., Currin-McCulloch, J., Packman, W. & Erdman, P. Disenfranchised guilt—pet owners’ burden. *Animals* **12**, 1690 (2022). 35804588 10.3390/ani12131690 PMC9264879

[93] Zerbe, W. J. & Paulhus, D. L. Socially desirable responding in organizational behavior: A reconception. *AMR* **12**, 250–264 (1987).

[94] El-Alayli, A., Lystad, A. L., Webb, S. R., Hollingsworth, S. L. & Ciolli, J. L. Reigning cats and dogs: A pet-enhancement bias and its link to pet attachment, pet-self similarity, self-enhancement, and well-being. *Basic Appl. Soc. Psychol.* **28**, 131–143 (2006).

