# Supplementary material for: Perceived costs and benefits of companion dog keeping based on a convenience sample of dog owners
Source: Sci Rep. 2025 Jan 21;15:2515. doi: 10.1038/s41598-025-85254-1 (PMC11751109; doi:10.1038/s41598-025-85254-1)
Supplement: Supplementary file 2 — Supplementary Information 2. [file 41598_2025_85254_MOESM2_ESM.docx]

**Supplementary Material S1**

Original questionnaire (English translation)

**Demographic data**

1. How old are you (years)?
2. What is your sex?

- Male
- Female
- Other:

1. What is your nationality?

[list of nationalities]

1. Where do you currently live?

- Large metropolitan area (population size is above 500 000)
- Small metropolitan area (population size is between 200 000 and 500 000)
- Medium-size urban area (population size is between 50 000 and 200 000)
- Small urban area (population size is below 50 000)
- Rural area, village (population size is below 2 000)

1. What describes best your current professional status?

- I am working full time
- I am working part time
- I am self-employed
- I am on long-term sick leave/maternity leave
- I am unemployed/looking for a job
- I am a stay-at-home parent
- I am a student
- I am retired
- Other:

1. What is your highest level of education?

- Primary school not completed
- Completed primary school
- Vocational training without a baccalaureate
- Graduate school
- Higher vocational or technical education
- Bachelor's degree (BSc) from a college or university
- Master's degree (MSc) from a college or university
- Higher education with an academic degree (PhD, etc.)

1. What would you say about your financial status?

- You have no financial problems
- You get by, with some budgeting
- You are just able to make both ends meet
- From month to month you have financial difficulties
- You live in hardship

**Dog Experience**

1. The following statements are about the potential benefits and costs of dog ownership. While some of them are clearly an advantage/disadvantage, other statements could be viewed either as an advantage or as a disadvantage, depending on who fills it in.

Therefore, each statement is presented on a **Big disadvantage…Neutral… Big advantage scale**.

Please try to rate each statement on this this scale based on what it means to YOU PERSONALLY, and not based on what it means to society in general.

Please try to answer even if you have no direct experience as a dog owner. If a statement is neither an advantage or a disadvantage for you, or is not applicable to your situation, please rate it as neutral (0).

- 3 = Big disadvantage -2 -1 0 = Neutral 1 2 3 = Big advantage

Dogs can cause allergies

Dogs need the love and affection of their owner(s)

Having a dog can have an influence on people’s quality of sleep

Having a dog can encourage people to be more physically active

Dogs can cause harm to other animals

Dogs can bring mess and dirt into the house

Having a dog can give people a reason to get up in the morning

Having a dog can give people the feeling of belonging to a community

Having a dog can help people to learn responsibility

The care of a dog can be expensive (e.g. food, vet bills, toys, brushes…)

Having a dog can lead to conflicts with other people (non-owners, neighbors, etc.)

Having a dog can make it difficult to find an appropriate place of living

When on vacation, a solution to care for the dog may be needed

Dogs can help their owner(s) to get through difficult situations in life

Dogs can keep children company

Having a dog can have an influence on their owners’ daily routine

Dogs need their owner(s) to take care of them

Dogs need to be trained and educated

Dogs can be noisy and barky

Dogs can contribute to a better family cohesion

Having a dog can facilitate interactions with other people (e.g. during walks, training)

Time must be devoted to the dog on a daily basis

Dogs can brighten one’s life

Having a dog can be emotionally challenging (e.g. in case of illness, impairments that come with ageing, grief)

Dogs’ disobedience can generate feelings of frustration, stress, and anger

Rescuing or adopting a dog can be a way to help another being in need, to contribute to a better world

Dog owners can feel ashamed or uncomfortable because of the (problematic) behavior of their dog

Dogs usually have a shorter lifespan than their owner(s)

Dogs can give their owner(s) a sense of security and stability in life

Having a dog can be a spiritual experience, and it could contribute to people’s self-growth

Dogs can be loyal and can provide unconditional love towards their owner(s)

Dogs can damage the property of their owner(s)

People can share fun moments of play and laughter with their dog

1. According to you, what is the biggest benefit of having a dog?
2. According to you, what is the biggest cost of having a dog?
3. Do you currently own a dog?

- Yes
- No

**About your dog...**

1. In total, how many dogs do you currently own?

From now on, if you have more than one dog, please focus on the one **you feel closest to**.

1. What is your dog’s name?
2. Sex of the dog

- Intact male
- Intact female
- Neutered male
- Neutered female

1. Please indicate the breed of your dog:

- Mixed breed/Unknown
- Other:

1. Date of birth of the dog (if you don’t know the date, please indicate its presumed age in the next question)
2. How old is your dog (presumed age, in years)?
3. How old was your dog when it joined your household?

- < 8 weeks old
- Between 8-12 weeks old
- Between > 12-16 weeks old
- Between > 4-6 months old
- Between > 6-12 months old
- Between > 1-5 years old
- Between > 5-10 years old
- More than 10 years old

1. Where did you obtain your dog from?

- From breeder
- From nonregistered breeder
- Bred by me/born at my place
- From shelter, animal rescue organization or dog breed rescuer
- From family/acquaintance
- Online (ad posted by a private individual)
- I found her/him on the streets

1. In your household, who usually takes care of this dog?

- Primary you
- Care is shared
- Primary another household member
